# Thinking styles and doctors' knowledge and behaviours relating to acute coronary syndromes guidelines

**DOI:** 10.1186/1748-5908-3-23

**Published:** 2008-04-25

**Authors:** Ruth M Sladek, Malcolm J Bond, Luan T Huynh, Derek PB Chew, Paddy A Phillips

**Affiliations:** 1 Flinders University, Sturt Road, Bedford Park, South Australia, 5042, Australia; 2 Flinders Medical Centre, Flinders Drive, Bedford Park, South Australia, 5042, Australia

## Abstract

**Background:**

How humans think and make decisions is important in understanding behaviour. Hence an understanding of cognitive processes among physicians may inform our understanding of behaviour in relation to evidence implementation strategies. A personality theory, Cognitive-Experiential Self Theory (CEST) proposes a relationship between different ways of thinking and behaviour, and articulates pathways for behaviour change. However prior to the empirical testing of interventions based on CEST, it is first necessary to demonstrate its suitability among a sample of healthcare workers.

**Objectives:**

To investigate the relationship between thinking styles and the knowledge and clinical practices of doctors directly involved in the management of acute coronary syndromes.

**Methods:**

Self-reported doctors' thinking styles (N = 74) were correlated with results from a survey investigating knowledge, attitudes, and clinical practice, and evaluated against recently published acute coronary syndrome clinical guidelines.

**Results:**

Guideline-discordant practice was associated with an experiential style of thinking. Conversely, guideline-concordant practice was associated with a higher preference for a rational style of reasoning.

**Conclusion:**

Findings support that while guidelines might be necessary to communicate evidence, other strategies may be necessary to target discordant behaviours. Further research designed to examine the relationships found in the current study is required.

## Background

Clear gaps remain between the best available scientific evidence and practice in a range of clinical disciplines [1,2], including cardiology [3-6]. Most research into reducing such gaps has been empirical and without reference to theory, and it has been argued that medicine now needs to look to other disciplines [7,8], including psychology [9], for relevant theories. How humans think and make decisions is important in understanding behaviour, and thus in the current context, an understanding of cognitive processes among physicians may inform our understanding of behaviour in relation to evidence implementation strategies in cardiology.

While ideas about logic and decision-making have been of interest since Aristotle, the concerted focus on decision-making began after World War II [10]. Developments coincided with a changing focus from a predominantly behaviourist perspective to an emerging cognitive framework for understanding human behaviour. Behaviourism was the dominant psychological framework that prevailed throughout the early and mid 1900s, emphasizing outward, observable behaviour as the only valid scientific area of psychological research [11]. Cognitive psychology, with its focus on internal processes and mental limitations [12], heralded twentieth century research into human thought and reasoning. In contrast to behaviourists, cognitive psychologists viewed inner processes that were not directly measurable as central to understanding human behaviour. To ignore thoughts and perceptions is to ignore what makes us human [13]. Cognitive-Experiential Self Theory (CEST) emerged in the 1970s as a global theory of personality that married the behaviourists' and cognitive psychologists' positions by linking cognitive processing with behaviour [14].

As a personality theory, CEST offers a framework in which to understand behaviour [15]. It proposes that we have four equally important needs that drive behaviour: to maximise pleasure and minimise pain, for relatedness, to maintain stability and coherence, and to enhance self esteem [16]. Behaviour is viewed as a compromise between these needs, each serving as a check and balance against the other. Each person constructs theories of reality in order to make life as emotionally satisfying as possible. Beliefs about reality are held in two cognitive systems, the rational and experiential, and through these (see Table 1), people adapt and make sense of their world [17]. Reviews have found strong support for the existence of these systems [18-21]. While differences exist between different models, it is generally agreed that there are strong familial resemblances between them. Most posit two cognitive modes of information processing that are in constant operation as we reason. One mode is described using terms such as rational, conscious, deliberate, slow, rule-based, and analytic. For example, learning to change gears in a car might be described as initially demanding on the rational mode for a novice driver. The other mode has been described as experiential, unconscious, automatic, fast, recognition-primed, and intuitive. An experienced driver rarely thinks deliberately about changing gears; the behaviours generally happen automatically.

**Table 1:** Comparison of the experiential and rational systems according to Cognitive-Experiential Self Theory

**Experiential**	**Rational**
Holistic	Analytic
Emotional; pleasure-pain oriented (what feels good)	Logical; reason oriented (what is sensible)
Associationistic connections	Cause and effect connections
Outcome oriented	Process oriented
Behaviour mediated by vibes from past experience	Behaviour mediated by conscious appraisal of events
Encodes reality in concrete images, metaphors, and narratives	Encodes reality in abstract symbols, words and numbers
More rapid processing oriented toward immediate action	Slower processing oriented toward delayed action
Slower to change; changes with repetitive or intense experience	Changes more rapidly; changes with speed of thought
More crudely differentiated; broad generalization gradient; categorical thinking	More highly differentiated; dimensional thinking
More crudely integrated; dissociative, organized in part by emotional complexes (cognitive affective modules)	More highly integrated
Experienced passively and preconsciously; seized by emotions	Experienced actively and consciously; in control of our thoughts
Self evidently valid; "Seeing is believing"	Requires justification via logic and evidence

Source: Epstein S: **Cognitive-Experiential Self-Theory of personality**. In: *Personality and social psychology*. Edited by Theodore Millon and Melvin J Lerner. New York: Wiley; 2003. [Irving B Weiner (Series Editor): *Handbook of psychology, vol 5.]*. Copyright ^© ^2003. Reprinted with permission of John Wiley & Sons, Inc.

These two modes of cognitive processing are well represented in over 40 years of research in medicine, spanning decision-making, diagnostic reasoning, problem solving, and clinical reasoning [22]. For example, medicine has often been referred to as an art and a science (*i.e.*, experiential and rational) [23,24]. Clinical expertise has been viewed as being stored in cognitive structures such as illness stories and schema, which enable rapid and non-analytical processing for most decisions (*i.e.*, experiential), while novices focus on biomedical knowledge, pathophysiology, and the use of causal models to make decisions, a slower and more analytical process (*i.e.*, rational) [25,26]. Arguments have been made about the relative supremacy of clinical versus statistical prediction (*i.e.*, experiential versus rational) [27-29].

According to CEST, the experiential system automatically interprets and organises experience, regulating most behaviour [30], but behaviour is assumed to be the product of both modes that interact simultaneously and sequentially in both directions [17]. Both modes may influence the other in negative and positive ways. The experiential mode can bias the rational by making quick cognitions that are incorrect or biased (impulsive thoughts or behaviour). Yet it also offers the rational mode access to novel information (*e.g.*, creative ideas). The rational mode is able to correct the experiential system (*e.g.*, understanding that impulsive behaviour may be counterproductive and thus resisting it) and can be taught to understand its operations and potential biases. Through repetition, rational mode activities may become proceduralised, and thus shift to the control of the experiential mode. This makes adaptive sense, as well-rehearsed thoughts and actions thus use less cognitive resources. To exist with a rational system alone would make even simple tasks, such as crossing a road, so demanding that we might never leave the curb [16].

The relative dominance of these two modes is influenced by a range of dispositional (individual) and situational (environmental) factors. The corrective operations of the rational are known to be impaired by time pressure, involvement in a concurrent cognitive task, diurnal rhythm, and mood [16]. Rational processing has been positively correlated with intelligence and exposure to statistical training [31]. Emotional arousal and experience are thought to influence the operations of the experiential system [16], as is mood [32], problem characteristics, decision characteristics, decision-making context, and lastly, but of most importance to the current paper, personal dispositions [33,34]. The ability and preference for using the two processing modes are believed to be relatively stable dispositions: need for cognition reflects the tendency to engage in and enjoy rational processing, and faith in intuition reflects the tendency to engage in and enjoy experiential processing [35]. We refer to these as 'thinking styles'. It has been shown that thinking styles make a unique contribution aside from intelligence to performance on a range of tasks [20,36-39].

According to CEST, thinking styles may influence an individual's receptivity to different messages [17]. This is therefore inherently of interest to those designing strategies to inform and encourage healthcare workers to change their behaviour. Matching messages to personal characteristics ('message matching') has been investigated elsewhere within the context of persuasive communication in health promotion [40-42]. Increased message effectiveness has been shown by matching messages to personality types [43], locus of control [44], self efficacy [45], attributional style [46], self schema [47], individuals' risk perception and willingness to seek information [48], and importantly, need for cognition [49,50].

A desirable stage of research prior to the experimental investigation of implementation strategies based on the CEST is an initial consideration of whether relationships between thinking styles and guidelines exist where they might be expected to do so. We hypothesised that higher need for cognition and/or lower faith in intuition would be associated with 1) awareness of the recently published guidelines; 2) knowing the topics included in the guidelines; 3) correctly answering questions about topics covered in the guidelines; and 4) higher need for cognition and/or lower faith in intuition would be associated with an overall higher self-reported estimate of guideline-concordant clinical practice. We also wished to investigate whether doctors with higher faith in intuition and/or lower need for cognition would be more likely to estimate guideline-discordant clinical practice overall, and across eight specific clinical scenarios. Table 2 provides a summary of the thinking styles assumed by CEST, and Table 3 summarises the current hypotheses in relation to these thinking styles.

**Table 2:** Rational Experiential Inventory in relation to Cognitive-Experiential Self Theory

	**Two Reasoning Systems proposed by Cognitive-Experiential Self Theory**	
	**Rational** (slow, deliberative, reflective)	**Experiential** (fast, automatic, reflexive)
	
Name of thinking style associated with each system and measured by the Rational Experiential Inventory	Need for Cognition	Faith In Intuition
Available scores using the Rational Experiential Inventory	Need For Cognition – total Need For Cognition – ability subscale Need For Cognition – favourability subscale	Faith in Intuition – total Faith in Intuition – ability subscale Faith in Intuition – favourability subscale

**Table 3:** Summary of hypotheses

	**Hypothesised relationship with need for cognition and/or faith in intuition**
	
**KNOWLEDGE**	
1) awareness of the recently published guidelines	Higher need for cognition and/or Lower faith in intuition (Hypotheses 1 – 4)
2) knowing the topics included in the guidelines	
3) correctly answering questions about topics covered in the guidelines	
**CLINICAL PRACTICE**	
4) overall higher estimate of guideline-concordant clinical practice	Higher faith in intuition and/or Lower need for cognition (Hypotheses 5 – 6)
5) overall higher estimate of guideline – *dis*cordant clinical practice	
6) higher estimate of guidelines *dis*cordant clinical practice across eight clinical scenarios	

## Methods

### Setting

An independent study of doctors' knowledge and behaviour in relation to the new National Heart Foundation of Australia/Cardiac Society of Australia and New Zealand Guidelines for the Management of Acute Coronary Syndromes 2006 (referred to as the Physician Guidelines Study) [51] conducted as an adjunct to the Australian Collaborative Acute Coronary Syndromes Prospective Audit [52] provided a timely opportunity to concurrently measure thinking styles.

### Participants

A sample of 84/91 (92.3%) medical personnel responded to the current study, from an underlying pool of respondents in the Physicians Guidelines Study (response rate 39.2%). These included consultant general physicians, consultant cardiologists, registrars, residents, interns, and private cardiac specialists with an active clinical role caring for patients with acute coronary syndromes. Only eight females responded, while two respondents did not indicate gender. For homogeneity, the final sample was restricted to the 74 male respondents, who had a mean age of 42.8 years (SD = 10.7).

### Design

The study was approved by the Flinders Clinical Research Ethics Committee. A questionnaire measuring thinking styles was included in the Physician Guidelines Study. Surveys were first mailed on 12 July 2006, with two follow-up contacts approximately three and six weeks later.

### Measures

The Rational Experiential Inventory (REI) reliably measures an individual's preference for two thinking styles: need for cognition (rationality) and faith in intuition (experientiality) [35]. Each construct has its own subscales relating to self-stated ability to think in each style (ability) and reliance and enjoyment on each type of thinking (favourability). The REI comprises 40 questions with five-point response scales (20 each for need for cognition and faith in intuition, with 10 items each for the subscales of ability and favourability). All scores are averaged to provide variables ranging from one to five, with a higher score reflecting a greater tendency to endorse the construct measured. The current sample provided internal reliabilities (Cronbach's α) of 0.84 (total need for cognition), 0.76 (need for cognition: ability), 0.74 (need for cognition: favourability), 0.91 (total faith in intuition), 0.84 (faith in intuition: ability), and 0.87 (faith in intuition: favourability). Preliminary analyses demonstrated that, consistent with CEST, there were only modest associations between need for cognition and faith in intuition scores, thus supporting their consideration as two separate, independent constructs (maximum *r *= -0.18, *p *= 0.130 for total scores).

The relevant data used from the Physician Guidelines Study comprised six questions [Additional file 1] that evaluated knowledge, attitudes, and clinical behaviours in relation to the recently published National Heart Foundation of Australia/Cardiac Society of Australia and New Zealand Guidelines for the Management of Acute Coronary Syndromes 2006 (the guidelines). These were publicly available three months prior to the survey (17 April 2006) [51]. Participants were asked to identify the correct publication date of the guidelines (Question One – Awareness), and to indicate which of three topics were covered (Question Two – General Knowledge). Detailed knowledge of topics covered in the guidelines was assessed using ten specific questions (Question Three – Topic Knowledge). Correct answers were tallied to provide a maximum score of 10. Respondents were then asked to assess, using five-point Likert scales, how often they based their practice on clinical guidelines (Question Four – Concordant Behaviour), and did not give their patients guideline-recommended care because it differed from what they had always done (Question Five – Discordant Behaviour). Finally, participants assessed the percentage of their patients who were given guideline-concordant treatment for eight specific clinical scenarios (use of aspirin, clopidogrel, beta blockers, calcium channel blockers, ACE inhibitors, statin therapy, early invasive strategy in high risk non-ST-elevation acute coronary syndromes and reperfusion therapy). Responses were averaged to give a mean percentage score, the inverse of which represented discordant practice (Question Six – Discordant Practice Rate).

### Statistical analysis

De-identified Physician Guidelines Study data were matched with responses to the REI. Analyses were conducted using SPSS Version 14.0 for Windows using Pearson's point-biserial correlation coefficients and Pearson's Product Moment correlation coefficients as appropriate, with statistical significance for all analyses set at *p *<0.05 (1-tailed). The effect of age was partialled out of all analyses given an unpublished earlier study suggested it had negative associations with faith in intuition.

## Results

Descriptive data are summarised in Table 4. The majority of respondents were aware that the guidelines were published in 2006 and that the three topics of unstable angina, non-ST-elevation myocardial infarction (NSTEMI), and ST-elevation myocardial infarction (STEMI) were covered. Only a small number achieved a maximum score (10/10) to knowledge based questions, although most agreed or strongly agreed that they often based their practice on clinical guidelines (68/74, 91.9%). Approximately half (38/74, 51.4%) also indicated that there are times when they do not give guideline-suggested care because it differs from their usual practice. Doctors' overall mean discordant practice rate was 27.40% (SD = 8.3%).

**Table 4:** Descriptive data for key study variables

Rational-Experiential Inventory (Mean, SD)	
Need for cognition (total score)	3.93 (0.37)
Need for cognition (ability)	4.04 (0.34)
Need for cognition (favourability)	3.82 (0.48)
Faith in intuition (total score)	3.05 (0.53)
Faith in intuition (ability)	3.27 (0.51)
Faith in intuition (favourability)	2.83 (0.64)
Acute Coronary Syndromes Knowledge and Behaviours	
Q1. Awareness (n/N, % correct answers) *Correctly knows year of publication*	50/74 (67.6)
Q2. General Knowledge (n/N, % correct answers) *Correctly knows topic coverage*	59/74 (79.7)
Q3. Topic Knowledge (Mean, SD) * Number of correct responses (max. 10)*	7.08 (1.35)
Q4. Concordant Behaviour (Mean, SD) * I often base my practice on clinical guidelines*	4.24 (0.64)
Q5. Discordant Behaviour (Mean, SD) * There are times when I do not give my patients guideline suggested care as the guidelines differ from what I have always done previously*	2.77 (0.94)
Q6. Discordant Practice Rate (Mean, SD) For patients under my care, the percentage with an acute coronary syndrome *who have no contraindications and are discharged on [treatment] *is approximately ...	27.40 (8.25)

Table 5 presents correlation coefficients between the variables of interest. Neither need for cognition nor faith in intuition scores were significantly related to correctly knowing the date of guideline publication (Physician Guidelines Study Question One) or scores relating to specific topic content knowledge of the guidelines (Physician Guidelines Study Question Three). Correctly knowing the topics covered in the guidelines (Physician Guidelines Study Question Two) was unrelated to need for cognition scores. However, knowledge of topic coverage was associated with lower faith in intuition scores (total and favourability subscale). Further, higher scores on need for cognition (total and ability subscale) were significantly associated with greater agreement with the statement "I often base my practice on clinical guidelines" (Physician Guidelines Study Question Four).

**Table 5:** Summary of correlations* relating to study hypotheses

	**Faith in Intuition**			**Need For Cognition**		
	
**ACS Knowledge and Behaviours**	**Ability**	**Favour-ability**	**Total**	**Ability**	**Favour-ability**	**Total**
Q1. Awareness	0.04	-0.02	0.01	0.05	0.05	0.06
*Correctly knows year of publication*	*p *= 0.373	*p *= 0.445	*p *= 0.470	*p *= 0.341	*p *= 0.337	*p *= 0.323
Q2. General Knowledge	-0.18	-0.20	-0.21	0.14	0.11	0.14
*Correctly knows topic coverage*	*p *= 0.057	*p *= 0.047	*p *= 0.041	*p *= 0.115	*p *= 0.135	*p *= 0.117
Q3. Topic Knowledge	-0.09	-0.08	-0.09	0.13	0.13	0.14
*Number of correct responses (max. 10*)	*p *= 0.233	*p *= 0.245	*p *= 0.222	*p *= 0.143	*p *= 0.138	*p *= 117
Q4. Concordant Behaviour	0.02	0.03	0.02	0.28	0.19	0.25
*I often base my practice on clinical guidelines*	*p *= 0.447	*p *= 0.389	*p *= 0.407	*p *= 0.009	*p *= 0.058	*p *= 0.008
Q5. Discordant Behaviour	0.20	0.29	0.27	-0.16	-0.10	-0.14
*There are times when I do not give my patients guideline suggested care as the guidelines differ from what I have always done previously*	*p *= 0.050	*p *= 0.007	*p *= 0.012	*p *= 0.092	*p = *0.208	*p *= 0.129
Q6. Discordant Practice Rate**	-0.27	-0.25	-0.28	-0.04	-0.08	-0.07
For patients under my care, the percentage with an acute coronary syndrome *who have no contraindications and are discharged on [treatment] *is approximately ...	*p *= 0.011	*p *= 0.020	*p *= 0.009	*p *= 0.373	*p *= 0.268	*p *= 0.293

*All probabilities are 1-tailed

** Q6: A positive correlation denotes a concordant practice rate; by default a negative correlation denotes discordant practice.

Higher faith in intuition (all scores) was significantly related to higher levels of agreement with the statement "there are times when I do not give my patients guideline-suggested care as the guidelines differ from what I have always done previously" (Physician Guidelines Study Question Five). Higher faith in intuition scores (all scales) was also associated with a higher average self-stated guideline discordant practice across eight different clinical scenarios concerning the use of: aspirin, clopidogrel, beta blockers, calcium channel blockers, ACE inhibitors, statin therapy, early invasive strategy in high risk non-ST-elevation acute coronary syndromes (NSTEACS), and reperfusion therapy (Physician Guidelines Study Question 6).

## Discussion

Doctors with a higher preference for the rational mode identified their practice as more guideline-concordant. A higher preference for the experiential mode was associated with guideline-discordant behaviours. However, a higher preference for the rational mode of reasoning was not significantly related to awareness or detailed knowledge of the guidelines. Only a lower preference for the experiential mode was associated with correctly knowing the topic coverage.

The key findings relate to the relationships between thinking styles and self-reported guideline concordance and discordance, although admittedly the effect sizes are small (r^2 ^range, 4.00% to 8.40%). Conversely, we must caution that these results have been observed using a very small sample, potentially leading to Type II errors. That is, additional, although even smaller, significant effects may have been missed due to a lack of power. Nevertheless, the available data demonstrated that guideline concordance (Physician Guidelines Study Question Four) was associated with a higher preference for the rational mode, and guideline discordance (Physician Guidelines Study Question Six) with the experiential mode.

There are several explanations for these findings. First, it may be that thinking styles are associated with perceived, and not real behaviour. Without objective data about an individual doctor's clinical practice, it is difficult to confirm or reject this possibility. Self-report is noted to be a particular methodological challenge for implementation research [53]. Second, it is possible that certain thinking styles are associated with a desire to present the self in a professionally appropriate manner. These possible explanations highlight the need for future research into thinking styles to be related to real-life individual clinical behaviours, observed and measured through processes other than self-report.

However, a third explanation is that thinking styles are indeed related to practice. While the association between the rational mode and concordance became non-significant when doctors were questioned less directly about guideline concordance and more specifically about their practices (Physician Guidelines Study Question Six), the relationship between the experiential mode and guideline discordant practices endured. The meaning of these observations can be understood by reference to a theoretical framework such as CEST.

Recall that CEST assumes a dual processing model of reasoning. That is, every person has their own unique preference for both the rational mode and the experiential mode. The current findings suggest that regardless of an individual's preference for the rational mode, it is their preference for experiential reasoning that may more readily influence their practice. This is consistent with an assumption in the CEST that most behaviour is regulated in the experiential mode. Findings suggest that while guidelines may communicate the necessary evidence, their distribution alone is unlikely to easily override experienced clinicians' practices. This is consistent with the evidence-to-date that simple dissemination of guidelines is a relatively unsuccessful implementation strategy.

CEST is more than simply a model of reasoning. Indeed, as a personality theory, it not only attempts to explain behaviour, but explicitly suggests strategies that may lead to changes in behaviour. The tenet of effective behavioural change according to CEST is that it must occur in the experiential mode. Three ways of producing change in the experiential mode have been identified [17]. First, the rational system can correct and train the experiential (an insight approach). Second, the experiential mode can be influenced through strategies such as the use of narratives, associations, metaphor, and fantasy, to which the experiential is known to be sensitive. Third, emotionally corrective experiences can be provided (cognitive-behavioural approaches). Numerous strategies based on these approaches could be tested experimentally. As an example, individualised audit and feedback of guideline concordance/discordance to physicians is one specific strategy consistent with an insight approach, where the rational mode (data about their actual practices) is used to correct the experiential mode (perceptions about their actual practices).

In summary, while the current study has presented an interesting set of data, it involved only a small and select group of clinicians. Therefore, prior to further speculation about potential interventions based on CEST, our results need to be replicated in other, preferably larger, studies comprising more representative samples.

## Conclusion

This study investigated thinking styles and awareness, knowledge, and behaviours of male doctors in relation to newly published acute coronary syndrome guidelines. Higher preference for an experiential mode of reasoning was associated with self-reported guideline discordance Higher preference for a rational mode of reasoning was associated with self-reported guideline use in relation to practice overall. These findings support that while guidelines might be necessary to communicate evidence, other strategies may be necessary to target discordant behaviours. Further research designed to test the relevance of CEST to clinician behaviour, that may replicate the current findings, is required.

## Competing interests

This study was supported by an unconditional research grant from Sanofi-Aventis. The funding agreement ensured the authors' independence in designing the study, interpreting the data, writing, and publishing the report.

## Authors' contributions

RMS, PAP and MJB conceived and designed this study, with contributions from LTH and DPBC. RMS and LTH undertook all data acquisition. RMS and MJB undertook the primary data analysis and interpretation. RMS drafted the paper and all authors were involved in its revision and final approval for publication.

## Supplementary Material

Additional file 1 Questions used from Physician Guidelines Study. This questionnaire was used to measure the knowledge, attitudes, and behaviour of doctors caring for patients with acute coronary syndromes in relation to recently published clinical guidelines. Click here for file
