# Supplementary material for: Thinking styles and doctors' knowledge and behaviours relating to acute coronary syndromes guidelines
Source: Implement Sci. 2008 Apr 25;3:23. doi: 10.1186/1748-5908-3-23 (PMC2386502; doi:10.1186/1748-5908-3-23)
Supplement: Additional file 1 — Questions used from Physician Guidelines Study. This questionnaire was used to measure the knowledge, attitudes, and behaviour of doctors caring for patients with acute coronary syndromes in relation to recently published clinical guidelines. [file 1748-5908-3-23-S1.doc]

**Questions used from the Physician Guidelines Study**

**Question 1.** Current Guidelines for the Management of Acute Coronary Syndrome were most recently published in:

 1999

 2000

 2001

 2002

 2003

 2004

 2005

 2006

 Due 2007

*[Correct response is 2006]*

**Question 2.** Current guidelines for the management of Acute Coronary Syndromes
 published by the National Heart Foundation and the Cardiac Society of
 Australia and New Zealand cover (please tick all boxes that apply):

 Unstable Angina

 NSTEMI

 STEMI

*[Correct response is to tick all three topics]*

**Question 3.** Please consider the following statements and mark only those with which you **strongly** agree.

 An elevated cardiac troponin (I or T) is diagnostic of cardiac chest pain

 An elevated CK-MK is diagnostic of cardiac chest pain

 With the advent of cardiac troponins, CK levels do not need to be measured

 An elevated cardiac troponin is a useful discriminator for risk of further
 events in patients presenting with suspected unstable angina*

 The ECG is the sole teat required to select patients for emergency
 reperfusion (thrombolytic therapy or direct PCI)*

 Exercise treadmill testing for unstable angina patients judged to have
 intermediate risk should be delayed until the result of cardiac troponin taken
 8 hours following initial chest pain*

 All patients presenting with chest discomfort should have an ECG
 completed within five minutes of arrival at a medical facility*

 Intermediate risk patients need to be treated with heparin unless reclassified
 into a low risk group

 Streptokinase is the reperfusion modality of choice in Aboriginal patients

 In patients with unstable angina and NSTEMI, randomized studies have
 shown no definite reduction in mortality with the use of beta blockers*.

*[* Indicates correct responses]*

**Question 4.** I often base my practice on clinical guidelines.

Strongly agree Agree Neutral Disagree Strongly agree

    

**Question 5.** There are times when I do not give my patients guideline suggested care as the
 guidelines differ from what I have always done previously.

Strongly agree Agree Neutral Disagree Strongly agree

    

**Question 6.** The following rating scale was provided for the 8 parts of question 6.
 *[100% represents maximal guideline concordance].*

|  |  |  |  |  |  |  |  |  |  |
| --- | --- | --- | --- | --- | --- | --- | --- | --- | --- |

0% 20% 40% 60% 80% 100%

**Question 6.1 (Aspirin)**

For patients under my care, the percentage with an acute coronary syndrome *who have no contraindications and are discharged on aspirin* is approximately …

**Question 6.2 (Clopidogrel)**

For patients under my care, the percentage with an acute coronary syndrome *who have no contraindications and are discharged on clopidogrel* is approximately …

**Question 6.3 (Beta Blockers)**

For patients under my care, the percentage with an acute coronary syndrome *who have no contraindications and are discharged on beta blockers* is approximately …

**Question 6.4 (Calcium Channel Blockers)**

For patients under my care, the percentage with an acute coronary syndrome *who have no contraindications and are discharged on calcium channel blockers* is approximately …

**Question 6.5 (Angiotensin Converting Enzyme (ACE) Inhibitors)**

For patients under my care, the percentage with an acute coronary syndrome *who have no contraindications and are discharged on an ACE Inhibitor* is approximately …

**Question 6.6 (Statin Therapy)**

For patients under my care, the percentage with an acute coronary syndrome *who have no contraindications and are discharged on statin therapy* is approximately …

**Question 6.7 (Early Invasive Strategy in High Risk NSTEACS)**

For patients under my care, the percentage who present with high risk NSTEACS and who are provided with early (within 48 hours) invasive management is approximately …

**Question 6.8 (Reperfusion Therapy)**

For patients under my care, the percentage with STEMI (who present within 12 hours and have no contraindications) and are given reperfusion therapy is approximately …
